# Metagenomic evidence for sulfur lithotrophy by Epsilonproteobacteria as the major energy source for primary productivity in a sub-aerial arctic glacial deposit, Borup Fiord Pass

**DOI:** 10.3389/fmicb.2013.00063

**Published:** 2013-04-22

**Authors:** Katherine E. Wright, Charles Williamson, Stephen E. Grasby, John R. Spear, Alexis S. Templeton

**Affiliations:** ^1^Department of Geological Sciences, University of Colorado at BoulderBoulder, CO, USA; ^2^Department of Civil and Environmental Engineering, Colorado School of MinesGolden, CO, USA; ^3^Geological Survey of Canada, Natural Resources CanadaCalgary, AB, Canada

**Keywords:** arctic, free energy, Epsilonproteobacteria, lithotrophy, metagenome, photosynthesis, sulfur, *Sulfurovum*

## Abstract

We combined free enenergy calculations and metagenomic analyses of an elemental sulfur (S^0^) deposit on the surface of Borup Fiord Pass Glacier in the Canadian High Arctic to investigate whether the energy available from different redox reactions in an environment predicts microbial metabolism. Many S, C, Fe, As, Mn, and ${\text{NH}}_{4}^{+}$ oxidation reactions were predicted to be energetically feasible in the deposit, and aerobic oxidation of S^0^ was the most abundant chemical energy source. Small subunit ribosomal RNA (SSU rRNA) gene sequence data showed that the dominant phylotypes were *Sulfurovum* and *Sulfuricurvum*, both Epsilonproteobacteria known to be capable of sulfur lithotrophy. Sulfur redox genes were abundant in the metagenome, but *sox* genes were significantly more abundant than reverse *dsr *(dissimilatory sulfite reductase)**genes. Interestingly, there appeared to be habitable niches that were unoccupied at the depth of genome coverage obtained. Photosynthesis and ${\text{NH}}_{4}^{+}$ oxidation should both be energetically favorable, but we found few or no functional genes for oxygenic or anoxygenic photosynthesis, or for ${\text{NH}}_{4}^{+}$ oxidation by either oxygen (nitrification) or nitrite (anammox). The free energy, SSU rRNA gene and quantitative functional gene data are all consistent with the hypothesis that sulfur-based chemolithoautotrophy by Epsilonproteobacteria (*Sulfurovum* and *Sulfuricurvum*) is the main form of primary productivity at this site, instead of photosynthesis. This is despite the presence of 24-h sunlight, and the fact that photosynthesis is not known to be inhibited by any of the environmental conditions present. This is the first time that *Sulfurovum* and *Sulfuricurvum* have been shown to dominate a sub-aerial environment, rather than anoxic or sulfidic settings. We also found that Flavobacteria dominate the surface of the sulfur deposits. We hypothesize that this aerobic heterotroph uses enough oxygen to create a microoxic environment in the sulfur below, where the Epsilonproteobacteria can flourish.

## INTRODUCTION

Microbes use a wide range of redox reactions to obtain energy for growth and therefore have a significant impact on the biogeochemical cycling of elements including carbon, nitrogen, and sulfur (Falkowski et al., 2008). Free energy calculations using geochemical analyses of an environment demonstrate that the most energetically-favorable redox reactions vary depending on the local chemistry and temperature (Amend and Shock, 2001; Shock et al., 2005, 2010; McCollom, 2007; Amend et al., 2011). Such calculations can be used to assess the habitability of diverse environments on Earth, or other planetary bodies such as Mars and Europa (Hoehler, 2007). However, it is often difficult to determine which reactions are utilized by microbes in any given environment. Some studies have investigated this question by environmental analysis of the small subunit ribosomal RNA (SSU rRNA) gene (Macur et al., 2004; Spear et al., 2005; Costa et al., 2009; Gaidos et al., 2009; Vick et al., 2010). However, this analysis is limited by our incomplete knowledge of the types of energy metabolism used by each phylotype. Another option is to use the polymerase chain reaction (PCR) to amplify genes for enzymes known to catalyze energy-releasing redox reactions (Hall et al., 2008; Chen et al., 2009; Flores et al., 2011). This approach is also limited, as it will only detect genes for which appropriate primers are used. Next-generation sequencing of metagenomes which have been produced from shotgun libraries overcomes many of these limitations. This method enables sampling of the functional genes present in an environment without the need for primers. Metagenomic and metatranscriptomic studies that investigate the relationship between geochemistry, energy sources, and microbial function have already been carried out on a range of different sites including ocean environments (Walsh et al., 2009; Canfield et al., 2010), Yellowstone hot springs (Inskeep et al., 2010), acidic sulfur-rich cave biofilms (Jones et al., 2012), and hydrothermal vents (Xie et al., 2011; Brazelton et al., 2012). The discovery of “cryptic cycling” of sulfur in an ocean minimum zone, a process not predicted from environmental geochemical data (Canfield et al., 2010), demonstrated the power of combining metagenomic and geochemical analyses to improve our knowledge of microbial ecology.

**FIGURE 1:**
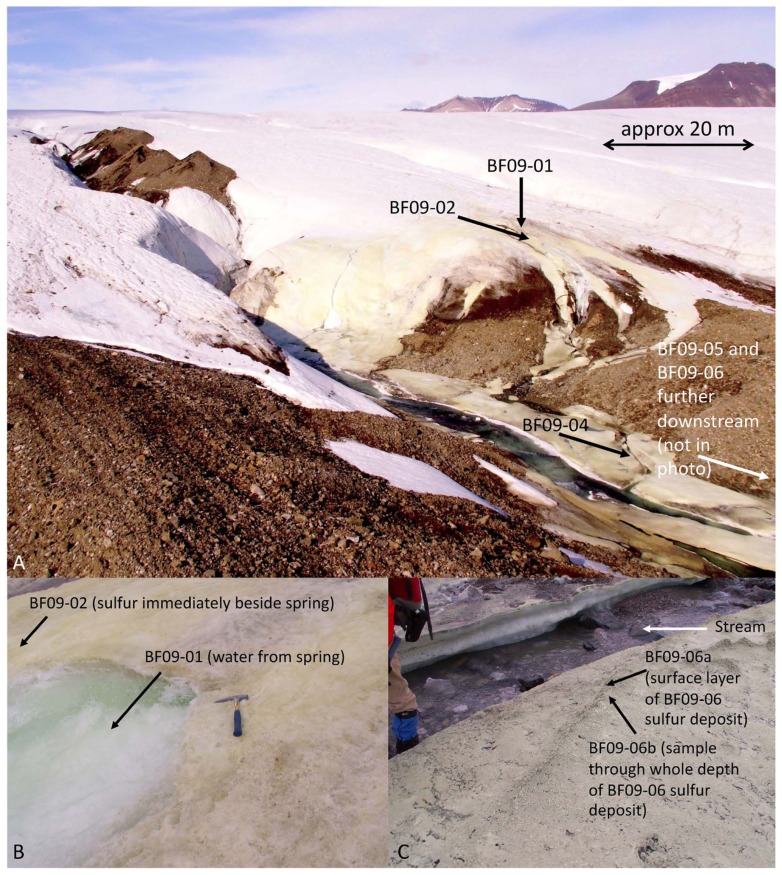
**Borup Fiord Pass Glacier**. **(A)** Overview of the field site in 2009. **(B)** The spring source (site BF09-01) and sulfur varnish beside the spring (site BF09-02). **(C)** Sulfur deposit BF09-06 from which DNA was extracted for the metagenome.

To date, no studies have combined a quantitative overview of energy availability with a quantitative overview of functional genes involved in energy-releasing reactions from the same environment. We have undertaken such a study at Borup Fiord Pass Glacier, Ellesmere Island, Nunavut, in the Canadian High Arctic (photo in **Figure 1A**; for a map of the area see **Figure 1** from Grasby et al., 2012). A cold sulfide spring flows over the glacier, surrounded by deposits of elemental sulfur (S^0^), gypsum, and carbonates on the surface of the ice that extend downstream for several hundred meters (Grasby, 2003; Grasby et al., 2003). It has not yet been determined whether the S^0^ is produced biologically, abiotically, or a combination of both. Borup Fiord Pass Glacier is an environment that is substantially different to sulfur-rich sites that have been the subject of previous metagenomic or metatranscriptomic studies. It is also an excellent terrestrial analog to consider the habitability of sites where subsurface waters penetrate icy overlayers in sulfur-rich environments on Mars or Europa (Gleeson et al., 2010, 2012; Grasby et al., 2012). The primary objective of this study was to undertake a comprehensive, robust, and quantitative study of whether the free energy of a specific environment predicts the microbial energy metabolisms that are utilized there. A secondary objective was to improve our understanding of which microbial groups are major players in cryogenic sulfur cycling, and which microbial sulfur redox reactions are likely to be significant in the glacial sulfur deposits at Borup Fiord Pass.

## MATERIALS AND METHODS

### SITE DESCRIPTION

Field work was carried out at Borup Fiord Pass Glacier, Ellesmere Island, Nunavut, in the Canadian High Arctic (81°N) in July 2009. A sulfide-rich spring rises through the glacier, near the toe, discharges from the surface of the glacier and flows into a supra-glacial meltwater stream. The spring rises in the same general area each year, but the exact site varies. The surface of the ice around the spring, and alongside its course, is covered with an elemental sulfur varnish. There is no volcanic or hydrothermal activity in the area that would explain the presence of the sulfur (Grasby et al., 2003) and evidence suggests that the spring is the result of a glacially-driven groundwater system (Grasby et al., 2012; Scheidegger et al., 2012).

### AQUEOUS AND MINERAL GEOCHEMICAL SAMPLING AND ANALYSIS

Measurements of temperature, pH, and oxygen concentration were made *in situ*. Spring water measurements were made with an Orion 5-star multimeter (Thermo-Scientific, USA), however, the multimeter would not give readings in the sulfur deposits. The pH values of the deposits were measured using colorpHast pH paper (EMD, Germany). Air temperature was measured using a hand-held thermometer (REI, USA). The temperature of the deposits was estimated to be between the temperature of the stream/ice and the temperature of the air. Oxygen concentrations in the sulfur deposits were not measured. Free energy calculations therefore used a ten-order of magnitude range of possible oxygen values, as described below. For aqueous geochemical analysis, spring water (sample BF09-01) was filter sterilized with a 0.2 μm filter and then injected into a sterile, argon-filled vial (to avoid oxidation of the sample) and maintained at approximately 4°C for 4½ days, during transport to the laboratory, then maintained at 4°C until analyzed. Ultra-pure nitric acid was added to one of the samples to avoid precipitation of aqueous cations. Samples of the sulfur deposits were collected using a sterile spatula and containers, frozen on collection, and then maintained frozen either in a freezer at -20°C, or packed in a cooler with freezer packs, during transport to the laboratory. At the laboratory samples were maintained frozen at -80°C until analyzed. Most of the sulfur was an extremely thin varnish (estimated to be no more than 1 mm thick) on the glacier surface (**Figure 1B** and **Table 1**). Sample site BF09-06 was a much thicker sulfur deposit located on ice beside the stream (**Figure 1C** and **Table 1**). Upon return to the laboratory, water was extracted from the BF09-06 sulfur deposit by thawing part of the sample, allowing the thawed sample to settle for approximately 5–10 min, and then removing the upper, water layer. This water was then filter sterilized using a 0.2 μm filter. Ultra-pure nitric acid was added to one of the water samples to preserve the solubility of cations. Both spring water and water extracted from the BF09-06 sulfur deposit were analyzed for sulfide, anions and cations. Sulfide was measured in the laboratory using the methylene blue method (Hach, USA), anions were measured using ion chromatography (Dionex), and cations were measured using mass spectrometry and inductively coupled optical emission spectroscopy. Fe^2+^ was assayed by the ferrozine method (Stookey, 1970). Ammonium was assayed using the phenol hypochlorite method (Weatherburn, 1967). Sulfide was also measured gravimetrically. At the site spring water was filter sterilized and cadmium acetate was immediately added to precipitate the sulfide, which was later filtered and weighed at the laboratory. Mineralogy of the sulfur deposit was determined by X-ray diffraction (XRD). Total carbon content of the BF09-06 deposit was measured by the Carla-Erba combustion method. Total dissolved organic carbon (DOC) measurements of the spring water, and the water extracted from the BF09-06 deposit, were made with a Shimadzu TOC-V CSN Total Organic Carbon Analyzer. Alkalinity was measured by end-point titration to pH 4.5 (Langmuir, 1997).

**Table 1:** Location of sample sites and description of samples.

Sample site	Description	Location	pH	Temperature
BF09-01	Water	Spring source	7.37 ± 0.14	-0.3°C
BF09-02	Sulfur varnish. Continuous layer of sulfur estimated to be 1 mm thick.	On top of ice immediately beside spring source.	6.0 ± 0.5	0°C (estimated, from the fact that deposit was in direct contact with ice)
BF09-04	Sulfur varnish. Continuous layer of sulfur estimated to be 1 mm thick.	On top of ice beside stream. Estimated to be 75 m from spring source.	7.0 ± 0.5	0°C (estimated, from the fact that deposit was in direct contact with ice)
BF09-05	Sulfur varnish. Continuous layer of sulfur estimated to be 1 mm thick.	On top of ice beside stream. Estimated to be 110 m from spring source.	7.0 ± 0.5	0°C (estimated, from the fact that deposit was in direct contact with ice)
BF09-06	Continuous layer of sulfur on top of ice. Sulfur deposit estimated to be 15 cm thick at deepest point. Sample BF09-06a was taken from the surface layer of the deposit and sample BF09-06b was taken through the whole depth of the deposit.	On top of ice beside stream. Estimated to be 300 m from spring source.	6.5 ± 0.5	0–5°C (estimated, from the facts that deposit was in direct contact with ice, and air temperature was 5°C)

### FREE ENERGY CALCULATIONS

The results of the geochemical analyses were used to calculate the energy potentially available from a range of redox reactions that could take place within the BF09-06 sulfur deposit, by the equation:

$$\Delta G_{r}=\Delta G_{\text{r}}^{ο}+RT\text{In}Q=RT\text{In(}Q/K)$$

where *G*_r_ is the free energy, $G_{\text{r}}^{ο}$ is the standard free energy, *R* is the universal gas constant, *T* is the temperature in Kelvin, *Q* is the reaction quotient, and *K* is the equilibrium constant. Energies were calculated at 0°C using values for the equilibrium constant (*K*) for each reaction at 0°C from The Geochemist’s Workbench software (v9.0, Aqueous Solutions, University of Illinois, USA) supplemented where necessary with thermodynamic data from Amend and Shock (2001). Concentrations of each ion were taken from the BF09-06 geochemical analysis. Where chemical species were below the detection limit of our instruments a range of concentrations was used to model ionic species (from 10^-12^ M, as being effectively zero, to the detection limit as a maximum). For As and Mn, only total element concentrations were measured. The Geochemist’s Workbench module Act2 was therefore used to determine the oxidized and reduced species of As and Mn most likely to be present at the pH of the sulfur deposit (pH 6.5). The maximum energy potentially available from oxidation was modeled by calculating the energy available if all the element was in the form of the reduced species, and the minimum energy was calculated using a concentration of 10^-12^ M for the reduced species. For atmospheric gases (O_2_, N_2_, and CO_2_), the maximum concentration possible in the sulfur deposit was calculated using the Henry’s Law constant for each gas at 0°C (Langmuir, 1997) assuming that the dissolved gases were in equilibrium with the atmosphere. The pO_2_ used to calculate the maximum possible dissolved oxygen was obtained using the equation:

$${\text{pO}}_{2}={\text{XO}}_{2}(\text{total atmosheric pressure}-{\text{pH}}_{2}\text{O})(\text{Langmuir,1997)}$$

where X is the atmospheric mole fraction of oxygen (0.21) and pH_2_O is the water vapor pressure (0.00611 bar at 1 bar total pressure and 0°C, Langmuir, 1997). The total atmospheric pressure used was 1 bar as the site elevation is close to sea level. To model energies from reactions that involve organic carbon the total DOC measured was assumed to be acetate. To calculate the maximum total energy available from reactions that involve S^0^ the total dry weight of BF09-06 was assumed to be S^0^, and to calculate the minimum energy it was assumed that only 10% of the dry weight was S^0^. Activities were calculated from the concentrations with the standard Debye–Hückel equation for ionic species, and the Setchenow equation for gases (Langmuir, 1997).

### SAMPLING FOR DNA EXTRACTION

Samples were taken from sulfur deposits immediately beside the spring source (site BF09-02), then moving progressively downstream (sites BF09-04, BF09-05, and BF09-06). Sampling was done with a sterile spatula that had been thoroughly washed in ultra-pure water (Milli-Q, Millipore, USA) prior to sterilization. Sample BF09-06a was taken from the surface layer of the BF09-06 deposit, while sample BF09-06b sampled the whole depth of the BF09-06 deposit. Samples were either (1) immediately immersed in 70% ethanol and maintained at 4°C during transport back to the laboratory (BF09-02, BF09-04, BF09-05, and BF09-06a) or (2) frozen on collection, and then maintained frozen either in a freezer at -20°C, or packed in a cooler with freezer packs, during transport (BF09-06b). All samples were maintained frozen at -80°C in the laboratory until DNA was extracted. Two aliquots of spring water (3 L each) were filtered *in situ* using a sterile 0.2 μm filter. The filters were preserved in 70% ethanol, maintained at 4°C during transport and then frozen at -80°C until DNA was extracted.

### DNA EXTRACTION AND PURIFICATION

DNA was extracted from one of the filters, and from samples BF09-02, 04, 05, and 06a, using a phenol–chloroform extraction as previously described (Dojka et al., 1998). DNA was extracted from the other filter using Trizol (Invitrogen, USA) to extract both RNA and DNA, following manufacturer’s instructions, except that a bead beating step (5 m/s for 45 s) was added at the start to lyse the cells. DNA for the metagenome was extracted from 65.822 g of the BF09-06b sample using the Powermax Soil DNA isolation kit (MoBio, USA) following the manufacturer’s instructions, except that at the final spin filter stage the extracts were combined to use only four filters instead of six, with an elution volume of 5 ml per filter. The eluant was concentrated using repeated ethanol precipitations and re-suspended in nuclease-free water (Sigma, USA). DNA was extracted from a further 5.335 g of the BF09-06b sample using a phenol–chloroform extraction (modified from Dojka et al., 1998). Briefly, 0.2–0.9 g of sample was suspended in buffer A (200 mM Tris [pH 8.0], 50 mM EDTA, 200 mM NaCl, 2 mM Na citrate, 10 mM CaCl_2_). Lysozyme was added to give a final concentration of 1 mg/ml and the sample was incubated at 37°C, inverting tubes to mix every 10 min, for 1 h. Proteinase K (to give 1 mg/ml) and sodium dodecyl sulfate (to give 0.3% wt/vol) were then added, and the sample was incubated at 37°C, inverting tubes to mix every 10 min, for a further hour. Tubes were centrifuged at 14,100*g* for 5 min. The supernatant was extracted first with 1 ml phenol:chloroform:isoamyl alcohol (25:24:1) then with 1 ml chloroform:isoamyl alcohol (24:1), followed by precipitation with sodium acetate (to give 0.3 M final concentration) and 100% cold isopropanol (equal volume to the aqueous phase). After precipitation the DNA pellet was washed twice with 70% ethanol and once with 100% ethanol, then re-suspended in sterile 10 mM Tris [pH 8.0]. The BF09-06b DNA samples from both the MoBio and phenol–chloroform extractions were combined and DNA of approximately 1.5 kb and longer was extracted from a 0.8% agarose gel using the E.Z.N.A. gel extraction kit (Omega Bio-Tek, USA) following the manufacturer’s instructions, with a final elution volume of 30 μl. The DNA was quantified using picogreen (Invitrogen, USA) and purity was assessed using a UV Nanodrop (Thermo Scientific, USA).

### SEQUENCING FOR SSU rRNA GENE ANALYSIS

DNA from BF09-02, BF09-04, BF09-05, BF09-06a, and BF09-06b was amplified with modified PCR primers 515F and 927R as previously described (Osburn et al., 2011). These primers have successfully amplified bacterial, chloroplast and archaeal SSU rRNA gene sequences in previous work (Osburn et al., 2011). The primers also contained a unique barcode for each sample. PCR amplicons were gel-purified using the E.Z.N.A. gel extraction kit (Omega Bio-Tek, USA) and then normalized using a SequalPrep Normalization Plate kit (Invitrogen, USA). Sequencing of the SSU rRNA amplicons was performed on a Roche pyrosequencer (Margulies et al., 2005) with FLX Titanium chemistry (Roche, Mannheim, Germany). The BF09-06b DNA was also amplified with the “universal” primers 515F and 1391R (Lane et al., 1985), gel-purified using the E.Z.N.A. gel extraction kit (Omega Bio-Tek, USA), cloned with the TOPO TA cloning kit for sequencing (Invitrogen, USA), and sequenced by Sanger sequencing. A nested PCR was used to amplify the DNA from the spring water (BF09-01); first the DNA was amplified with “universal” primers 515F and 1391R, gel-purified using the E.Z.N.A. gel extraction kit (Omega Bio-Tek, USA), then further amplified with primers 515F and 927R, and prepared for pyrosequencing as above. The primers 515F and 1391R have successfully amplified bacterial, archaeal, and eucaryal SSU rRNA gene sequences in previous studies (Ley et al., 2006; Spear et al., 2007).

### PYROSEQUENCING OF THE METAGENOME

A shotgun library was made from the BF09-06b DNA using the Rapid Library Preparation Method (Roche, Germany) and sequenced using a full plate on a Roche pyrosequencer with FLX Titanium chemistry (Roche, Germany).

### ANALYSIS OF SSU rRNA GENE PCR AMPLICON DATA

Small subunit ribosomal RNA PCR amplicon pyrosequencing data were analyzed with QIIME v1.5.0 (Caporaso et al., 2010). To exclude poor quality data, sequences with minimum average quality score less than 25, ambiguous bases, primer or barcode mismatches, or maximum homopolymer run greater than six nucleotides, were discarded. Only sequences between 410 and 470 nucleotides were used in the analysis as sequences that are significantly longer or shorter than the typical length for a sequencing run have been shown to be poor quality (Huse et al., 2007). The remaining sequences were denoised using flowgram clustering (Reeder and Knight, 2010). Primer sequences were removed using a custom script. Sequences were clustered into operational taxonomic units (OTUs) at 97% identity using UClust (Edgar, 2010) and the most abundant sequence in each cluster was chosen as the cluster representative sequence (rep seq) to assign taxonomy for the OTU. The same OTUs were used across all samples, so that rep seqs could come from any sample. Chimeras were identified by ChimeraSlayer (Haas et al., 2011) using the “gold” 16S NAST-aligned MicrobiomeUtilities database^1^ as the reference database, and chimeric sequences were discarded. Taxonomic classifications of pyrosequences were assigned with the RDP classifier (Wang et al., 2007) and the Greengenes taxonomy (DeSantis et al., 2006; OTU reference and utility files gg_otus_4feb2011 from http://greengenes.lbl.gov). For OTUs that represented more than 5% of the sequences for any sample, taxonomic identification at the Genus level was determined by using basic local alignment search tool (BLAST; Altschul et al., 1990) to compare the rep seq for that OTU against the National Center for Biotechnology Information (NCBI) nucleotide non-redundant (nt nr) database. The taxonomy of the Sanger sequences was also determined by using BLAST against the NCBI nt nr database. Reference sequences for *Sulfurovum*, *Sulfuricurvum*, and *Sulfurimonas* SSU rRNA genes (all >800 nt) were downloaded from NCBI to form the basis of the Epsilonproteobacteria phylogenetic tree. The rep seqs for the most abundant OTUs identified as *Sulfurovum*, *Sulfuricurvum*, and *Sulfurimonas* in the BF09-06b SSU rRNA gene data were BLASTed against the Borup Sanger sequences to find the best matches. Good matches were found for *Sulfurovum* (BF09-06_M97 at 100% identity to OTU 754 rep seq) and *Sulfuricurvum* (BF09-06_M102 at 99.46% identity to OTU 686 rep seq) but no match was found in the Borup Sanger data for the *Sulfurimonas* rep seq (OTU 742 rep seq). The Borup Sanger sequences BF09-06_M97 and BF09-06_M102 and the Epsilonproteobacteria reference sequences were aligned and masked using SSU-ALIGN (Narwocki, 2009). Maximum-likelihood trees were made with the online RAxML Black Box (Stamatakis, 2006; Stamatakis et al., 2008) using the gamma model of rate heterogeneity, and performing 100 bootstrap replicates. Pyrosequences (rep seqs for OTUs 754, 686, and 742) were aligned and masked using SSU-ALIGN (Narwocki, 2009), then added into the RAxML tree with pplacer (Matsen et al., 2010).

### METAGENOME SEQUENCE DATA ANALYSIS

The analysis was performed on unassembled reads. The MG-RAST (the metagenomics RAST) web-based analysis package version 3 (Meyer et al., 2008) was used to produce a quality-controlled database from the metagenome data by removing reads less than 75 bp in length, that contained 10 or more ambiguous bases in the sequence, or that were artificial duplicates created by the sequencing process (identified as reads which started at the same base and which had exactly the same sequence for the first 50 bases). Quality data on the metagenome before and after the quality control process are shown in **Table 2** below.

**Table 2:** Data on the number and quality of sequences in the metagenome.

	Initial metagenome database	Post quality control database
Number of sequences	1,238,751	957,074
Average length (bp)	530 ± 63	537 ± 37
Total bp	657,394,433	514,016,777
GC %	41 ± 9	41 ± 9
Predicted protein-coding sequences	Not applicable	823,237
Annotated protein-coding sequences	Not applicable	536,866

The quality-controlled database was analyzed to provide functional and phylogenetic information using the MG-RAST analysis pipeline, which uses the BLAST-like algorithm BLAT (Kent, 2002). We set a maximum e-value of 10^-10^ and a minimum percentage identity of 50% for functional gene annotation against the GenBank nr database. The MG-RAST software gives only approximate quantifications, and this can include counting the same metagenome sequence more than once in a result if there are two or more equally good top hits. Therefore, to produce accurate quantifications, the metagenome sequences that were recorded as hits against genes of interest were downloaded, and duplicate entries (i.e., the same metagenome sequence being recorded twice as a hit against the same gene) were removed manually to produce a quality-controlled abundance for the number of hits against each gene. As the metagenome DNA sequences were not the full gene length, longer genes would automatically get more hits than shorter genes, even if the two genes were present in the environment in the same numbers. The abundance of each gene was therefore normalized against gene length, by dividing the quality-controlled abundance by the gene length in kb, to produce an abundance per kb of gene (as described in Canfield et al., 2010). As genes with more than one copy per genome would also receive more hits than single-copy genes, the abundance per kb of gene was divided by the copy number per genome to give a value that could be used to compare directly the normalized relative abundances (NRA) of two genes within the metagenome, as a measure of the relative abundance of organisms possessing each gene within the environment. The gene lengths and gene copy numbers used for each gene were those from the organisms that accounted for the majority of the best hits for the metagenome sequences of that particular gene. As the metagenome had been prepared using random shearing to create a shotgun library, the relative abundance within the metagenome data was considered to be a reasonable assessment of the relative abundance of each gene within the environment. As the metagenome DNA was a random sample of the DNA in the environment, in order to determine whether differences in the NRA seen in the genes were statistically significant, the standard error (SE) of each NRA was calculated using the equation:

$${\text{SE}=[p(1-p)/n]}^{1/2}$$

where *p* is the proportion of the total results given for a particular NRA, and *n* is the total number of results (Gardner and Altman, 1986). Error bars for each sample were calculated at the 99% confidence level (±2.58 SE). This means that there is a probability of 0.99 that the NRA of a gene in the environment is included within the error bar range, so that NRA values that do not have overlapping error bars are significantly different at the *p* = 0.01 level. Only genes for metabolic reactions of interest were quantified. The SSU rRNA gene sequence data within the quality-controlled database were also analyzed using the MG-RAST analysis pipeline, setting a maximum e-value of 10^-10^ and a minimum percentage identity of 95% for taxonomic annotation against the Greengenes database. No sequences were assigned to more than one taxonomic group and duplicate sequences within the same taxonomic group were removed to avoid double-counting.

### GENOME COVERAGE

The estimated expected genome coverages for the most abundant organisms were calculated using the method set out in Whitaker and Banfield (2006). Briefly, this method estimates the proportion of the metagenome library (*P*_i_) derived from each species-level OTU by:

$$P_{\text{i}}=[G_{\text{i}}A_{\text{i}}]/\Sigma [G_{\text{i}}A_{\text{i}}]$$

where *G* is the genome size, and *A* is the abundance in the sample, of each species-level OTU. Abundances were taken from the proportion of total SSU rRNA reads for each species-level OTU, using the same OTUs as in **Figure 3B**. Genome sizes were taken from the genomes of sequenced organisms that were close relatives of each of the most abundant OTU phylotypes. These reference genomes were *Sulfurovum NBC37-1*, *Sulfuricurvum kujiense*, *Flavobacterium frigoris*, *Burkholderia pseudomallei*, and *Ralstonia picketti*. For the low abundance phylotypes the genome size was assumed to be 3 Mb. The mean expected coverage (*m*) of the genome from each species-level OTU is then given by:

$$m=P_{\text{i}}T/G_{\text{i}}$$

where *T* is the total number of base pairs in the post quality control metagenome library. A Poisson distribution for DNA sequencing is assumed, so that the estimated proportion of each genome that will not be sequenced is given by e^-*m*^ and therefore the estimated proportion of each genome that is included in the metagenome dataset is given by 1 -e^-*m*^.

### SEQUENCE DATA ACCESS

All the DNA sequences associated with this study are available on the NCBI website^2^. The metagenome sequences and the SSU rRNA gene PCR amplicon pyrosequences have been deposited in the NCBI Sequence Read Archive (NCBI SRA), grouped under BioProject PRJNA186721. The metagenome accession number is SRR765967 and the SSU rRNA gene PCR amplicon pyrosequences have accession numbers SRR654094–SRR654099. The SSU rRNA gene sequences obtained by Sanger sequencing have been deposited in GenBank, grouped together under PopSet 451617337, with accession numbers KC435454–KC435501. The metagenome sequences are also available on the MG-RAST website^3^ with reference number 4466433.3.

## RESULTS

### GEOCHEMISTRY

Aqueous geochemistry of the spring water and water extracted from the BF09-06 sulfur deposit is given in **Table 3** below.

**Table 3:** Geochemical analysis of the spring water (BF09-01) and water extracted from the glacial elemental sulfur deposit (BF09-06b) from which DNA was extracted and sequenced for the metagenome.

	BF09-01	BF09-06b	DL
Total Na (mM)	51.562	1.601	0.001
Total K (mM)	0.303	0.040	0.005
Total Mg (mM)	12.876	0.836	0.0003
Total Ca (mM)	17.306	10.921	0.003
Total Fe (μM)	5	1	0.215
Fe^2+ ^(μM)	BDL	BDL	5
${\text{NH}}_{4}^{+}$ (μM)	254	15	5
Total Mn (μM)	1	13	0.01
Total As (μM)	0.217	0.006	0.0001
Total Si (μM)	88	22	1.317
Total sulfide (mM)	4–6.3	BDL	0.005
${\text{SO}}_{4}^{2-}$ (mM)	13.029	9.381	2
${{\text{S}}_{2}\text{O}}_{3}^{2-}$(μM)	102	BDL	2
Cl^-^ (mM)	39.072	2.119	0.006
F^-^ (μM)	54	31	11
Br^-^ (μM)	33	BDL	1
${\text{NO}}_{3}^{-}$(μM)	3	13	8
${\text{NO}}_{2}^{-}$(μM)	BDL	BDL	11
${\text{PO}}_{4}^{3-}$ (μM)	BDL	BDL	2
Alkalinity (mg/L)	15	163	7.5 × 10^-4^
Total DOC (mg/L)	3.9	11.4	0.06
Total organic carbon (%w of solid)	–	0.12	0.04

The spring water had high levels of sulfide (4 mM measured gravimetrically and 6.3 mM by the methylene blue method) and also contained 13 mM sulfate and 102 μM thiosulfate. No sulfide or thiosulfate were detected in the sulfur deposit itself. Iron, manganese, ammonium, and arsenic were also detected in both the spring water and sulfur deposit. Nitrate was detected in the sulfur deposit and the spring water, and nitrite was also detected in the sulfur deposit. However, the spring water nitrate and sulfur deposit nitrite measurements were below the detection limit to which the ion chromatograph had been calibrated, and therefore these could not be considered accurate quantifications. XRD showed that the BF09-06 deposit was composed of S^0^ with a very small amount (estimated <3%) of gypsum.

### FREE ENERGY CALCULATIONS

**FIGURE 2:**
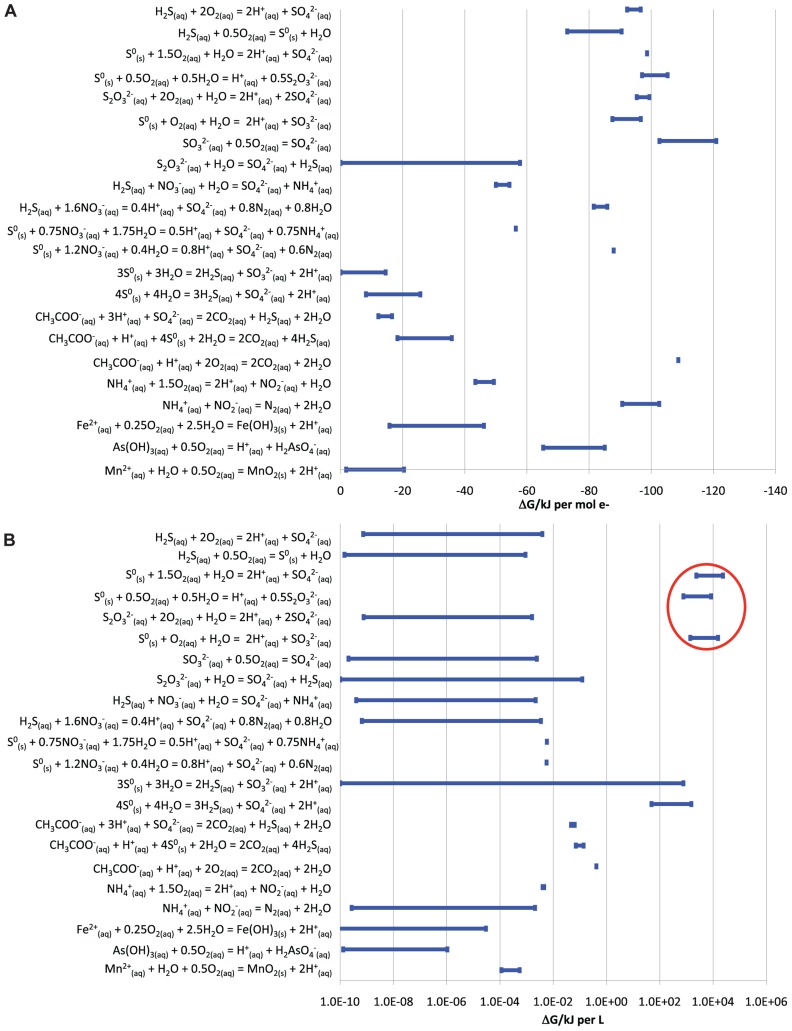
**Energy available from different redox reactions that could occur in the BF09-06 sulfur deposit**. Ranges in the amount of energy reflect the range of uncertainty for substances that could not be detected (up to a maximum of the detection limit used for the assay). **(A)** The energy available per electron transferred. **(B)** The total energy available from the same reactions in the BF09-06 sulfur deposit, taking into account the total amount of each reactant present. The energy available from the aerobic oxidation of S^0^ is ringed in red.

Many reactions could provide energy for microbial growth in the BF09-06 sulfur deposit. The most energy available per electron transferred was from the aerobic oxidation of reduced sulfur species (sulfide, S^0^, thiosulfate, or sulfite), aerobic oxidation of organic carbon, or anammox (**Figure 2A**). The maximum possible dissolved oxygen concentration in the sulfur deposit was calculated to be 4.4 × 10^-4^ M (14 mg/L). The maximum chemical activity of dissolved oxygen was calculated from the maximum concentration and was equal to 4.4 × 10^-4^. The Gibbs free energy calculations shown in **Figure 2A** used the maximum chemical activity of oxygen. As the sulfur deposit was in direct contact with the atmosphere it was considered feasible that oxygen concentrations could potentially be at the maximum concentration at the surface. However, as oxygen levels might decrease below the surface of the deposit, energies for oxidation of sulfur species were also calculated for a range of concentrations down to 10^-10^ M, which corresponded to a minimum chemical activity of 10^-10^. These reactions continued to be highly energetically favorable (-80 to -110 kJ per mole electrons transferred) even at these low oxygen levels. Comparison of the total energy available from different sources in the deposit showed that the energy available from aerobic S^0^ oxidation was greater than from any other measured source, including from the aerobic oxidation of organic carbon (**Figure 2B**). The results in **Figure 2B** assumed that oxygen could be resupplied by ongoing diffusion from the atmosphere into the sulfur deposit.

### IDENTITY OF MICROORGANISMS IN THE SPRING WATER AND SULFUR DEPOSITS

**FIGURE 3:**
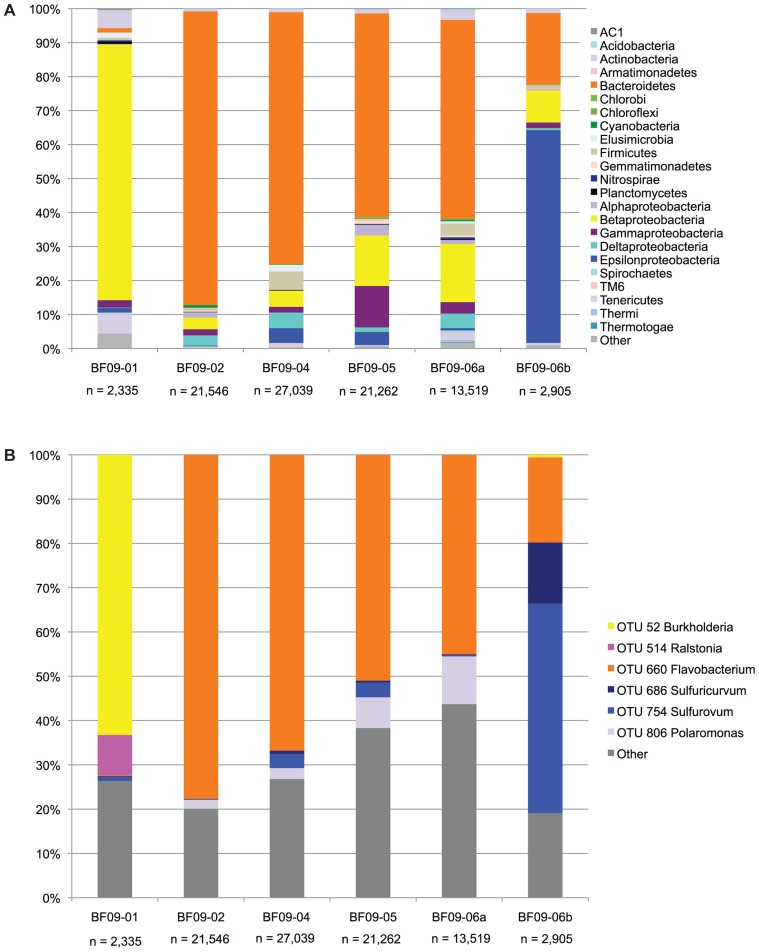
**SSU rRNA gene sequence data from the PCR amplicon library.** **(A)** The SSU rRNA data at the phylum level, except that the proteobacteria have been split into classes. Phyla which are less than 0.01% in all samples are included in “other.” **(B)** Each OTU representing more than 5% of any sample, with all other OTUs included in “other.”

The SSU rRNA gene PCR amplicon sequence data showed that the sulfide spring and glacial sulfur deposits were dominated by a few bacterial phylotypes (**Figure 3**).

In the spring water Burkholderiaceae (*Burkholderia* and *Ralstonia*) were strongly dominant. In BF09-02, BF09-04, BF09-05, and the BF09-06 surface sample (BF09-06a) *Flavobacterium* were dominant, but in the BF09-06 deposit as a whole (sample BF09-06b) Epsilonproteobacteria (*Sulfurovum* and *Sulfuricurvum*) were strongly dominant. For each of the *Flavobacterium*, *Sulfurovum*, and *Sulfuricurvum* a single species-level OTU (grouped at 97% SSU rRNA identity) comprised most of the sequences (**Figure 3B**). *Thiomicrospira* (Gammaproteobacteria) and *Thiobacillus* (Betaproteobacteria), other Genera known to be capable of sulfur lithotrophy, were present at significantly lower abundances (1.3 and 0.8% of BF09-06b, respectively). No eucaryal sequences and only four archaeal sequences were detected in the SSU rRNA pyrosequencing data (*n* = 88,606 across all samples). There were no eucaryal or archaeal sequences in the BF09-06b pyrosequencing data or the Sanger data (*n* = 50, data not shown).

**FIGURE 4:**
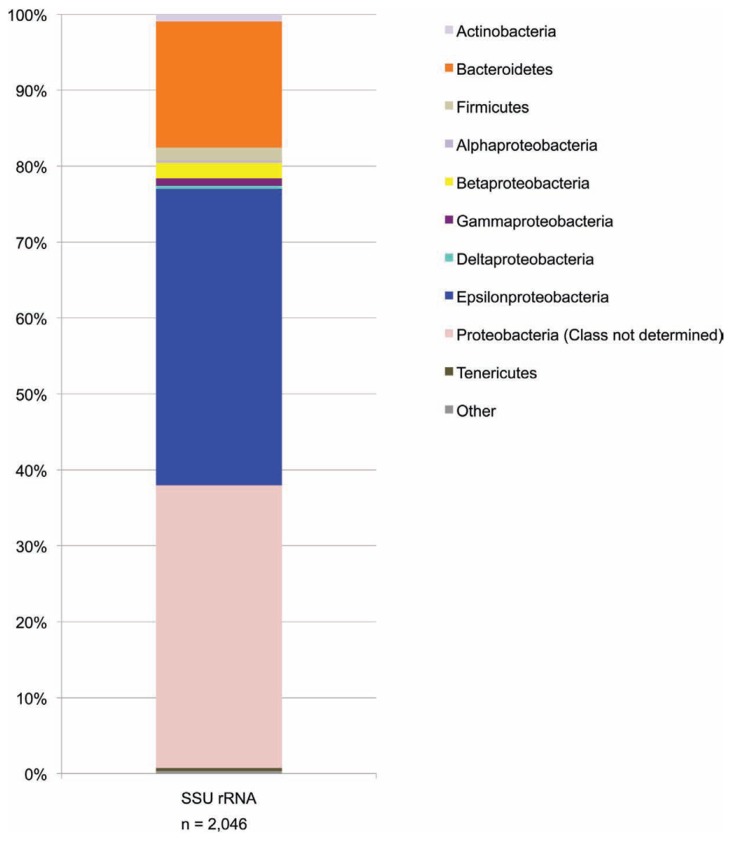
**SSU rRNA gene sequence data from the metagenome shotgun library**. Data are shown at the phylum level except that the proteobacteria have been split into classes. For some metagenome sequences the most closely related sequences in the Greengenes database were identified as proteobacteria but without specifying which Class. These are the sequences included in the “Proteobacteria (class not determined)” group. Phyla which represent less than 0.1% of sequences are included in “other.”

The SSU rRNA gene sequence data from the metagenome quality-controlled database (**Figure 4**), showed a consistent pattern with the SSU rRNA gene sequence PCR amplicon data (compare **Figure 4** with the BF09-06b data in **Figure 3A above**).

**FIGURE 5:**
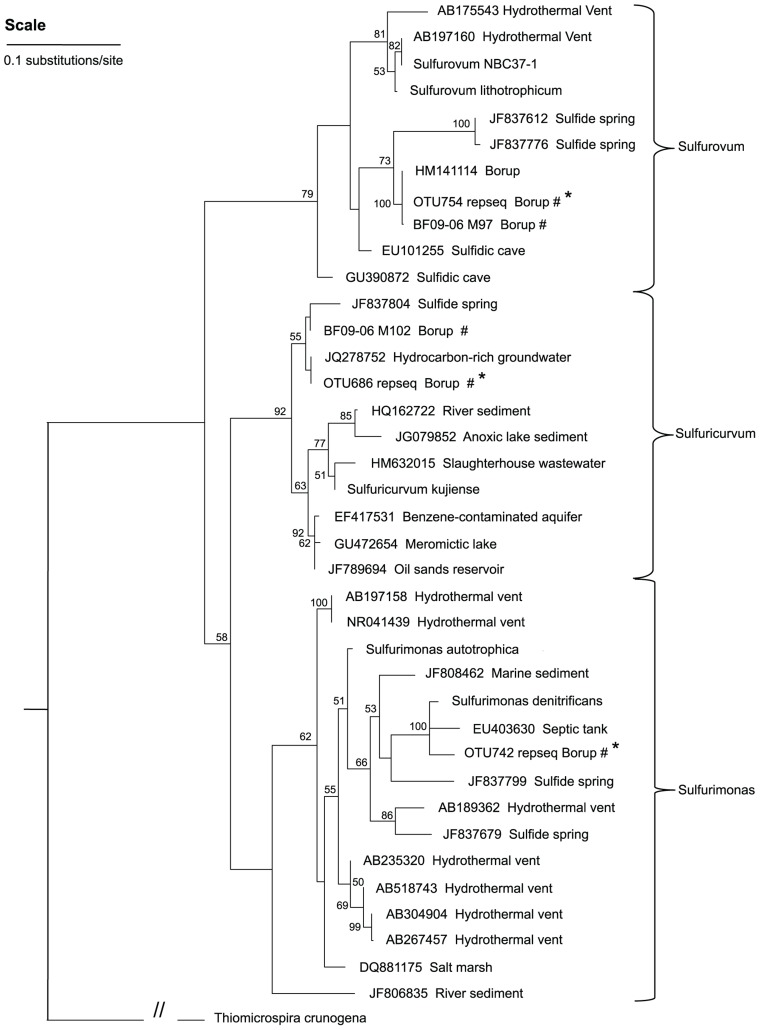
**A maximum-likelihood tree representing the phylogenetic relationships of the dominant Epsilonproteobacteria OTUs in the BF09-06 sulfur deposit to Epsilonproteobacteria reported from other sites**. Bootstrap values of less than 50 are not shown and these nodes are considered un-resolved. *Thiomicrospira crunogena *(Gammaproteobacteria) is the outgroup used to root the tree. Key: ^#^environmental sequences from this study; *shorter sequence from 454 sequencing.

A phylogenetic tree (**Figure 5**) showed that the dominant Borup *Sulfurovum* sp. and *Sulfuricurvum* sp. were closely related to sequences identified at thermal sulfide springs on Svalbard Island (Reigstad et al., 2011).

### GENOME COVERAGE

Our calculations estimated that the metagenome library contained full coverage of the genomes of the three most abundant organisms (although genomes were not assembled) but did not contain full coverage of lower abundance organisms (**Table 4** below).

**Table 4:** Estimated mean genome coverage, and percentage of genome included in the metagenome library, for the five most abundant species-level OTUs. The OTUs are the same as those shown in **Figure 3B**.

OTU	Mean genome coverage	Percentage of genome included in the metagenome
*Sulfurovum*	×40.94	100.00
*Sulfuricurvum*	×11.87	100.00
*Flavobacterium*	×16.51	100.00
*Burkholderia*	×0.54	41.46
*Ralstonia*	×0.06	5.78

### SULFUR REDOX GENES

**FIGURE 6:**
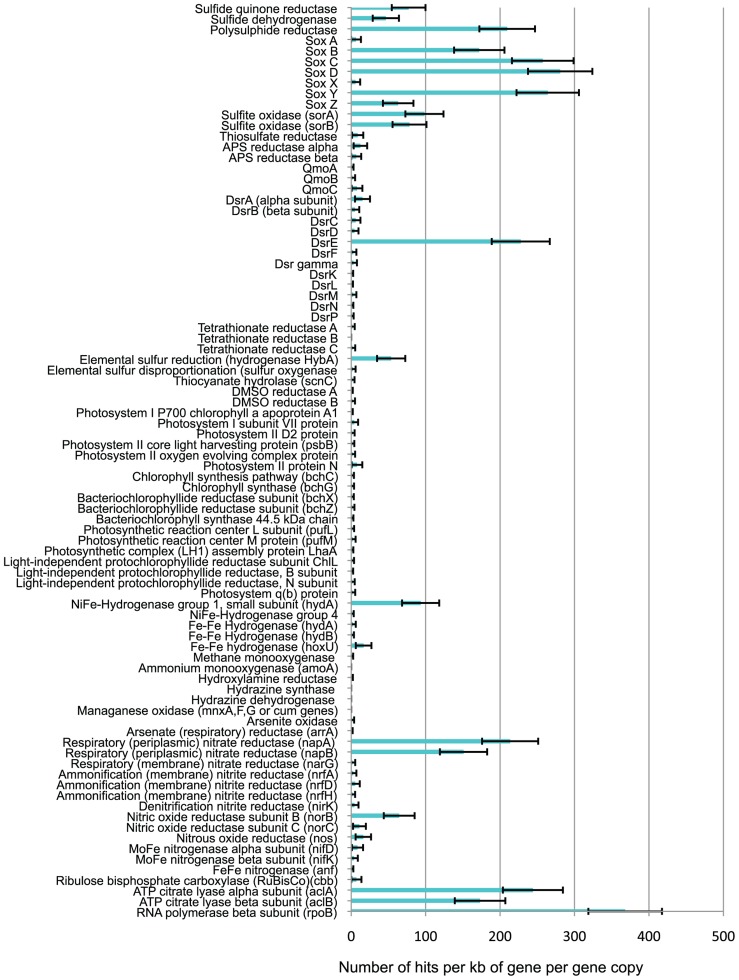
**Normalized relative abundance (NRA) of functional genes in the metagenome**.

The metagenome contained abundant sulfur redox genes (**Figure 6**), including genes for: sulfide quinone reductase (*sqr*) and sulfide dehydrogenase (*fcc*, *soxEF*, or *sud*) both involved in oxidizing sulfide to elemental sulfur; sulfur oxidation (Sox) proteins (*sox ABCDXYZ*), known to be involved in the oxidation of thiosulfate and also shown *in vitro* to have the capability to oxidize sulfide, elemental sulfur, and sulfite (Rother et al., 2001); sulfite oxidoreductase (*sor*), which oxidizes sulfite to sulfate; sulfur oxygenase reductase (also *sor* genes) involved in the disproportionation of elemental sulfur to sulfide and sulfite; thiosulfate reductase (*phs*), responsible for the disproportionation of thiosulfate to sulfide and sulfate; polysulphide reductase (*psr*), used to reduce polysulfides to sulfide; dissimilatory sulfite reductase (*dsr*), known to be involved in the reduction of sulfite to sulfide and vice versa; adenosine 5′-phosphosulfate (APS) reductase (*apr*) and quinone-interacting membrane-bound oxidoreductase (*qmo*) involved in the oxidation of sulfite to sulfate, and vice versa; tetrathionate reductase (*Ttr*), DMSO reductase (*dms*), thiocyanate hydrolase (*scn*), and elemental sulfur reductase (*HybA* hydrogenase; Friedrich et al., 2005; Stewart et al., 2011). However, the relative abundance of the genes present in the metagenome varied significantly. The *sqr*, sulfide dehydrogenase, *sox*, *psr*, sulfite oxidase, and *dsrE* genes were present in significantly higher relative abundance (NRA values of 47–281) than other sulfur redox genes, including the other *dsr *genes (NRA values of 1–15).

### OTHER ENERGY METABOLISM GENES

There were almost no photosynthetic genes present in the metagenome (**Figure 6**), or phototrophs within the SSU rRNA gene sequence data. This indicates that the sulfur deposits did not contain significant numbers of microbes capable of either oxygenic or anoxygenic photosynthesis, relative to the numbers of non-photosynthetic microbes. Significant numbers of hydrogenases were detected in the metagenome (**Figure 6**), the vast majority of which were NiFe-hydrogenases group 1 (*hydA*) which are respiratory hydrogenases allowing microbes to use hydrogen as an electron donor in redox reactions (Mulder et al., 2010). In addition, the metagenome contained sequences for the Fe–Fe hydrogenase (*hydA,B, hoxU*) typically used by microbes to generate H_2_ by using protons as terminal electron acceptors in their electron transport chains in order to allow respiration to continue in anoxic environments when no other suitable electron acceptor is present (Boyd et al., 2010). The metagenome contained large numbers of the respiratory (periplasmic) nitrate reductase (*nap*) genes (NRA > 150), indicating the genetic potential for nitrate respiration, but very few *nar* genes for membrane-bound respiratory nitrate reductase (NRA = 2). The metagenome contained genes for nitrite reductase (*nir*), nitric oxide reductase (*nor*), and nitrous oxide reductase (*nos*), involved in denitrification, but with higher NRA of *nor *and *nos* genes (NRA = 16–64) than nir genes (NRA = 4). Very few ammonification nitrite reductase (*nrf*) gene sequences were present (NRA = 2–5). No copies of the first gene in the aerobic oxidation of ammonium (*amoA*) were detected, and there was only one hit against hydrazine oxidoreductase (the second gene involved in ammonium oxidation to nitrite). No hydrazine synthase or hydrazine dehydrogenase genes (indicative of anammox) were found. Almost no arsenite oxidase (*aox*, *aro*, or *arx*) genes were found, and no manganese oxidation (*mnx* or *cum*) genes. Both reactions were potentially energetically favorable but as only total As and Mn concentrations were assayed, arsenite or reduced manganese may not have been present.

### CARBON AND NITROGEN FIXATION

The metagenome contained almost no RuBisCo (*cbb*) genes, indicative of carbon fixation via the Calvin–Benson cycle (**Figure 6**). There was a high relative abundance (NRA = 173–244) of ATP citrate lyase (*acl*) genes, indicative of carbon fixation via the reductive tricarboxylic acid (TCA) cycle (Hugler et al., 2005). Comparison of the NRA range for RNA polymerase B (*rpoB*), which is believed to be a single-copy gene in bacteria (Canfield et al., 2010), with the NRA of *acl* genes, demonstrated the *acl* genes are present in about 50–60% of all microbes at the site. Very few nitrogenase (*nif*, *anf*, or *vnf*) genes (NRA = 0–8) indicate very little ability to fix nitrogen.

### ORIGIN OF GENES

**FIGURE 7:**
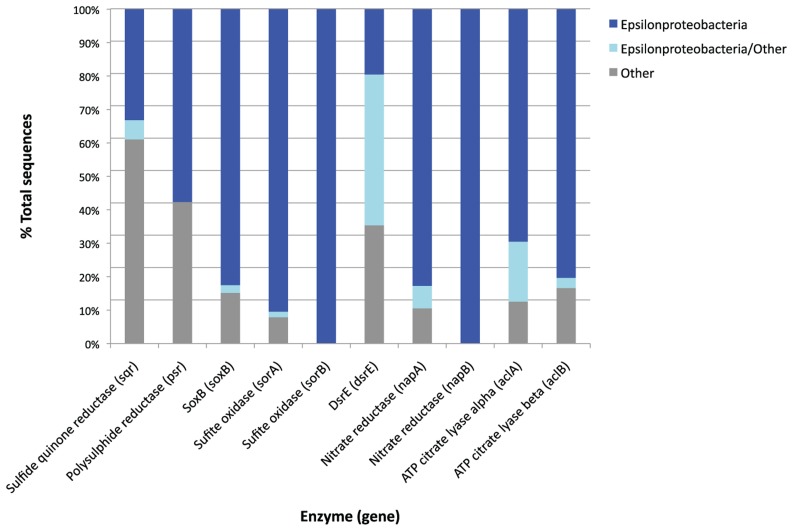
**Taxonomic origin of the best hits of metagenome genes which are found in high relative abundance produced by the MG-RAST analysis pipeline**. Where a metagenome sequence has two or more equally good best hits the MG-RAST analysis retains all the best hit results. The “Epsilonproteobacteria/other” category is sequences for which there were two or more best hits against the same functional gene, but from different phyla. The taxonomic origin of these sequences cannot therefore be clearly determined. The “other” category includes all the other phyla represented within the best hits for each gene, and differ from gene to gene.

The majority of the “best hits” for *soxB*, *psr*, *sor*, *acl*, and *nap* gene sequences were of epsilonproteobacterial origin (**Figure 7**). This is consistent with the fact that Epsilonproteobacteria dominated the BF09-06b SSU rRNA gene data.

## DISCUSSION

Our free energy calculations based on the geochemical analysis of the BF09-06 sulfur deposit, the SSU rRNA gene data and the data on functional genes found in the metagenome, strongly establish the hypothesis that the main energy source for primary productivity in the BF09-06 deposit is the oxidation of reduced sulfur species. The metagenome data show that the BF09-06 microbes have the genetic capability to oxidize sulfide through multiple oxidation reactions to sulfate. The free energy calculations (**Figure 2**) confirm that this is highly energetically favorable at every stage and that the oxidation of S^0^ provides the most energy.

We infer that *Sulfurovum* and *Sulfuricurvum* are the major primary producers in this sulfur-driven microbial ecosystem. All the sulfur redox genes that are present in high relative abundance are present in the genomes of sequenced representatives of these Genera,* Sulfurovum NBC37-1 *(NCBI accession number: NC_009663, Nakagawa et al., 2007) and *Sulfuricurvum kujiense* (NCBI accession number: NC_014762). This indicates the genetic capability to oxidize sulfide, thiosulfate and sulfite, and to reduce polysulfide. Cultured representatives of *Sulfurovum* and *Sulfuricurvum* have shown the ability to grow using energy from the oxidation of reduced sulfur species, including sulfide, S^0^, and thiosulfate (Kodama and Watanabe, 2003, 2004; Inagaki et al., 2004; Nakagawa et al., 2007; Yamamoto et al., 2010; Yamamoto and Takai, 2011) and to use the reductive TCA cycle to fix carbon (Kodama and Watanabe, 2004; Hugler et al., 2005; Nakagawa et al., 2007). It is therefore likely that the *Sulfurovum* and *Sulfuricurvum* present in the BF09-06 sulfur deposit also have these capabilities, although it is not clear which reduced sulfur species are being utilized or which sulfur redox genes are being expressed.

No sulfide was detected in the BF09-06 deposit, but the sulfide assay was not done until several days after sample collection, by which time any sulfide could have oxidized. Thiosulfate and sulfite were not detected either, but these species are often present below detection levels due to rapid cycling (Thamdrup et al., 1994; Zopfi et al., 2004). Both thiosulfate and sulfite will form by abiotic oxidation of sulfide (Zhang and Millero, 1993) and so would be generated if sulfide were present. Polysulfides will form abiotically when sulfide and S^0^ react, and are the main product of the sulfide quinone reductase oxidation of sulfide *in vitro* (Griesbeck et al., 2002). They would therefore probably also be present in the deposit if sulfide were there. The Borup *Sulfurovum* and *Sulfuricurvum* sp. could potentially be involved in cycling sulfur, by aerobic oxidation of sulfide to polysulfide using *sqr* genes, and then re-reducing the polysulfide to sulfide using *psr* genes if a suitable reductant is present. Organic carbon could, in theory, be used to reduce polysulfides, but culturing of *Sulfurovum *has not so far demonstrated this ability (Yamamoto et al., 2010).

S^0^ is abundant on the glacial surface, and aerobic oxidation of S^0^ was the most abundant energy source at the time of sampling, though it is important to note that the most important energy source over time may vary depending on the flux of sulfur species and other nutrients into the deposit. However, given the known abilities of cultured *Sulfurovum* and *Sulfuricurvum* to oxidize S^0^ it seems likely that the Borup *Sulfurovum* and *Sulfuricurvum* are oxidizing S^0^ to provide energy for primary productivity.

The genes involved in the oxidation of S^0^ are still not completely understood. The reverse *dsr* gene products are known to oxidize S^0^ as well as sulfide (Dahl et al., 2005, 2008) but these genes were not detected in significant quantities in the metagenome. The *sox* gene products have been shown to oxidize S^0^ *in vitro* (Rother et al., 2001) although to our knowledge it has not yet been proven that bacteria possessing the *sox* genes actually use them in this way *in vivo*. There are logistical challenges in enabling an intracellular enzyme complex to access an extracellular insoluble substrate like S^0^. An intriguing possibility for how the periplasmic Sox proteins might be able to access external S^0^ is provided by the high relative abundance of *dsrE* family genes in the metagenome, and their presence in the genomes of* Sulfurovum NBC37-1* and *Sulfuricurvum kujiense*, which do not contain the other *dsr* genes. In the full *dsr* complex, the *dsrE* gene product is one of those used to mobilize S^0^ that is inside the cell (Dahl et al., 2008). Our data therefore raise the question of whether the DsrE**family protein in Epsilonproteobacteria might be used to mobilize external S^0^ so that Sox**proteins can oxidize it.

It is not clear why the relative abundance of *sox* genes is so much higher than the relative abundance of reverse *dsr* genes. The total amount of energy potentially available from the oxidation of sulfide to sulfate is constant, whichever pathway is used. However, the amount of energy conserved from a reaction will not be equivalent to the total energy that is potentially available, as no enzyme is 100% efficient. Different enzymes may have different levels of efficiency (Sievert and Vetriani, 2012). Alternatively, the higher relative abundance of *sox* genes could simply be a consequence of better environmental fitness of the Epsilonproteobacteria compared to bacteria that use the *dsr* complex for other reasons, unrelated to energy.

The Sox protein complexes that have been studied comprise Sox A,B,C,D,X,Y,Z (Friedrich et al., 2005) but our results show that the relative abundance of *sox A*, *X*, and *Z* are lower than the relative abundances of the other *sox *genes, particularly for *sox* *A* and *X* (**Figure 6**). The SoxZ protein normally exists in a heterodimer with the SoxY protein, but it has been suggested that SoxY may sometimes form a homodimer (Friedrich et al., 2005) which could explain why the NRA of the *soxZ* gene is lower than that of the other *sox* genes. For *soxA* and *soxX *we suggest that the very low NRA is due to mis-annotation of two key genes in GenBank. The *Sulfurovum NBC37-1* genome does not contain an annotated *soxX *gene, but this *Sulfurovum* has been shown to possess an active Sox protein complex (Yamamoto et al., 2010). The *Sulfurovum NBC37-1* protein encoded at locus SUN_0497 is very closely related to the *Sulfuricurvum* SoxX protein (86.44% identity in the amino acid sequences with an e-value of 6e^-08^). Although the gene at SUN_0497 is annotated as a hypothetical protein we consider that it is extremely likely to be a *soxX* gene, and this is consistent with the conclusions of other researchers who have implicitly referred to this gene as a *soxX* gene (Yamamoto and Takai, 2011). Similarly, the *Sulfuricurvum kujiense* genome does not contain an annotated *soxA* gene, despite the fact that this *Sulfuricurvum* has been shown to be able to oxidize thiosulfate (Kodama and Watanabe, 2004). The *Sulfuricurvum kujiense* protein encoded at locus Sulku_0448 is closely related to the *Sulfurovum* SoxA protein (50% identity in the amino acid sequences with an e-value of 4e^-59^), and when we BLASTed this protein against the GenBank protein nr database all the best hits were also SoxA proteins. Given the evidence from culture studies, this protein is very likely to be a SoxA protein, but it is annotated as a hypothetical protein. As our analysis utilized the GenBank annotations, genes which were not annotated as *sox* genes were not included in the **Figure 6** *sox* gene data. Given the numerical dominance of *Sulfurovum* sp. and *Sulfuricurvum* sp. in our sample, we consider that the probable mis-annotation of these two genes explains their low relative abundance in **Figure 6**.

The metagenome contains very small numbers of genes known to be involved in reductive sulfur redox reactions (*dsr*, *apr*, and *qmo* genes) or the disproportionation of thiosulfate and S^0^ (*phs* and sulfur oxygenase reductase *sor* genes, respectively). Our free energy calculations (**Figure 2B**) indicate that S^0^ disproportionation could potentially yield significant energy for microbial growth in this environment, but the NRA of genes involved in S^0^ disproportionation is very low (**Figure 6**). We conclude that reductive and disproportionation sulfur redox reactions are unlikely to be as prevalent as the oxidative reactions in this environment. In contrast, reductive and disproportionation reactions have been shown to be significant components of sulfur cycling in marine sediments (Jørgensen and Nelson, 2004). A crucial caveat to our conclusion is that few S^0^ disproportionation genes have been characterized, so they may be less well represented in GenBank than other sulfur cycling genes. This could result in S^0^ disproportionation genes being under-represented in our analysis.

### NITRATE AND NITRITE RESPIRATION

The metagenome contains very high numbers of periplasmic nitrate reductase (*nap*) genes, illustrating that the microbial community in the sulfur deposit possesses the capability for nitrate respiration. Both the sequenced and cultured representatives of *Sulfurovum* and *Sulfuricurvum *are known to respire nitrate (Inagaki et al., 2004; Kodama and Watanabe, 2004; Nakagawa et al., 2007) suggesting that the Borup strains of these Genera also have this capability. The genes needed for denitrification and nitrate ammonification, the onward reduction of nitrite to nitrogen gas and ammonium, respectively (Zumft, 1997) are present in much lower numbers than the *nap* genes. Very little nitrate was detected in the deposit, and although a nitrite measurement was taken, it was below the lower limit of the standard curve used for the assay, and so cannot be considered reliable. Nitrate or nitrite could be used as significant oxidants by the microbial community if they were being replenished. The genes for nitrification (the aerobic oxidation of ammonium to nitrate via hydroxylamine and nitrite) were at extremely low relative abundance or not detected at all. Atmospheric deposition may be a possible nitrate source (Holtgrieve et al., 2011) and very low levels of nitrate have been detected in glacial run-off streams (0.03–0.15 ppm, S. Grasby, unpublished data) although these data are from a different year.

### OVERALL CONCLUSIONS ON METABOLIC PATHWAYS

**FIGURE 8:**
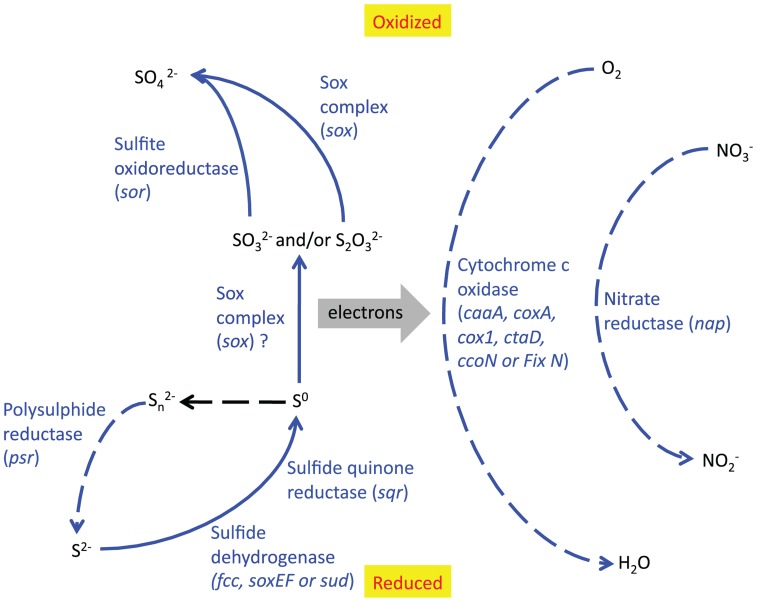
**Reaction pathways which are likely to be significant in the BF09-06 sulfur deposit, based on the relative abundance of functional genes**. Solid lines indicate oxidative reactions and dotted lines indicate reductive reactions. Blue lines indicate reactions that are catalyzed by enzymes whose genes are present in high relative abundance. The protein and gene names are written beside the relevant reaction. The black line is an abiotic reaction.

Taking into account the free energy calculations, SSU rRNA gene data and the relative abundance of functional genes in the metagenome, we conclude that several sulfur redox reactions may be significant in this environment (see **Figure 8**). DNA evidence only shows the genetic potential of microbes, not which genes are actively being used, so it is impossible to tell which of these reactions are actually being catalyzed by the bacteria in the sulfur deposit. However, our current interpretation of the integrated data is that the Borup *Sulfurovum* and *Sulfuricurvum* are oxidizing reduced sulfur species, in particular S^0^, using oxygen and possibly also nitrate, and that these reactions are being used to provide energy sources for carbon fixation. The numerical dominance of Epsilonproteobacteria in the SSU rRNA gene data suggests they are the main primary producers of this site. This is further supported by the fact that the majority of sulfur redox and carbon fixation genes appear to be of epsilonproteobacterial origin (**Figure 7**).

### SUB-AERIAL *SULFUROVUM* AND *SULFURICURVUM*

This is the first study in which either *Sulfurovum* or *Sulfuricurvum* have been shown to be dominant in a sub-aerial environment. In previous studies, *Sulfurovum* has been found in sulfidic environments including springs, hydrothermal vents, caves, sinkholes, and anoxic/sulfidic sediments (Nakagawa et al., 2005; Macalady et al., 2006, 2008; Porter and Engel, 2008; Borin et al., 2009; Han et al., 2009; Jones et al., 2010; Sahl et al., 2010; Ramos-Padrón et al., 2011; Reigstad et al., 2011; Handley et al., 2012) and it has been suggested that they preferentially colonize environments with high levels of sulfide and low levels of oxygen (Macalady et al., 2008; Jones et al., 2010). This accurately describes the spring water which we believe was the source of the organisms that seeded the glacial sulfur deposit, but it is not clear how well this describes the deposit itself as we were not able to determine a sulfide:oxygen ratio. Culturing studies of *Sulfurovum* demonstrates that it grows better in microoxic conditions than in a fully oxygenated environment (Yamamoto et al., 2010). The cultured *Sulfuricurvum* has also been shown to grow in anoxic or microoxic environments (Kodama and Watanabe, 2004) and environmental sequences are from anoxic, often sulfidic, sites (Watanabe et al., 2000; Kodama and Watanabe, 2003; Wagner et al., 2007; Haaijer et al., 2008; Chen et al., 2009; Gaidos et al., 2009). Our SSU rRNA gene data also show that *Flavobacterium* are more abundant in the surface part of the BF09-06 deposit than the Epsilonproteobacteria, and are by far the most abundant phylotype in the thin sulfur varnishes (BF09-02, BF09-04, and BF09-05). As *Flavobacterium* species have been characterized as aerobic heterotrophs we hypothesize that the aerobic metabolism of *Flavobacterium* at the surface of BF09-06 may be reducing the oxygen availability, creating a microoxic environment in the lower parts of the deposit, thus creating the conditions in which *Sulfurovum* and *Sulfuricurvum* can thrive. Variations in sulfide levels within the deposits could also potentially be a factor, since sulfide levels in the sulfur deposit could not be accurately measured. The low abundance of *Sulfurovum* and *Sulfuricurvum* in the much thinner sulfur varnish deposits support the hypothesis that they cannot thrive in a fully oxygenated environment.

### FLAVOBACTERIUM

The role of *Flavobacterium* in this system is not determined. *Flavobacterium* species are known heterotrophs, and there is energy available from organic carbon for growth. However, the relatively high level of *Flavobacterium* in the surface sulfur deposits raises the question of whether these microbes play a role in sulfur cycling as well. Microbial heterotrophic sulfur cycling has been shown to be significant in other environments (Mason and Kelly, 1988; Sorokin, 1996). *Flavobacterium* species have been shown to oxidize thiosulfate (Teske et al., 2000) and dimethyl sulfide (Green et al., 2011) in culture, although organic carbon was still required for growth. It is not clear why *Flavobacterium* is so dominant in the thin sulfur varnishes, and this remains a subject of investigation.

### MISSING METABOLISMS

A surprising feature of this microbial community is the fact that some energetically favorable reactions appear not to be utilized. There were almost no genes present for photosynthesis or ammonium oxidation despite the fact that these are energetically favorable. RuBisCo is used by many bacteria to fix carbon, including phototrophs, so its absence is also consistent with the lack of phototroph SSU rRNA genes or photosynthetic functional genes. Photosynthesis is commonly regarded as the dominant energy source for primary productivity in “light” environments. As our field site is at high latitude, at the time of sampling it had been under 24-h sunlight for 3 months, and the presence of plant life in the proglacial area proves that photosynthesis is energetically feasible. Other studies have also demonstrated that both oxygenic and anoxygenic phototrophs can grow in permanently cold and arctic conditions, although productivity may be lower than elsewhere (Hughes and Lawley, 2003; Roeselers et al., 2007; Simon et al., 2009; Ng et al., 2010; Schmidt et al., 2010). While sulfide is known to inhibit oxygenic photosynthesis (Oren et al., 1979; Miller and Bebout, 2004) some cyanobacteria readily utilize sulfide for anoxygenic photosynthesis (Castenholz and Utkilen, 1984; Cohen et al., 1986; Jørgensen et al., 1986). The possible presence of sulfide also does not explain the absence of green and purple sulfur phototrophs. These not only tolerate, but use, sulfide, and dominate other cold sulfidic sites (Douglas and Douglas, 2001; Ley et al., 2006; Klepac-Ceraj et al., 2012). While photosynthesis is known to be inhibited by temperatures above 72°C (Hamilton et al., 2012) that is clearly not an issue in this environment. The fact that there were a few SSU rRNA genes from phototrophs, and very small numbers of photosynthetic functional genes (only one or two sequences per gene) indicates that phototrophs are able to reach the site via global water and/or wind distribution systems, and other work has shown evidence of strong local aerial transport of cyanobacteria in the area (Harding et al., 2011). One possible hypothesis is that the relative absence of phototrophs is due to a combination of several factors that are present at this site: (1) the arctic setting allows only a short growth season, due to lack of light and extreme cold during winter; (2) the glacial surface appears to be a relatively pristine site, as it is not covered in soil, plant, or visible microbial growth, and (3) the spring emerges in a slightly different place each year, seeding the glacial surface with microbes from a dark subsurface environment. The large influx of subsurface organisms (unlikely to contain many phototrophs), together with an abundant energy supply for microbes capable of aerobic oxidation of reduced sulfur, may therefore allow for microbial growth that is more rapid than phototroph colonization via random atmospheric transportation. In hydrothermal vent environments Epsilonproteobacteria have been shown to be the most rapid initial colonizers, even though they are not necessarily dominant in the environment overall (López-Garcia et al., 2003; Nakagawa et al., 2005). This may help to explain why Epsilonproteobacteria are dominant in our sulfur deposits despite the fact that they are not dominant in the spring water. An alternative hypothesis for the lack of phototrophs is that they are excluded due to competition with other microbes present at the site. These hypotheses are not mutually exclusive. Further work is needed to determine the reasons why the relative abundance of phototrophs is so low. The role played by the Burkholderiaceae that dominate the spring water is also unknown.

The absence of ammonium oxidation is also intriguing. Ammonium oxidation by oxygen (nitrification) or nitrite (anammox) is energetically favorable according to our free energy calculations, and nitrification has been shown to be significant in a subglacial site (Boyd et al., 2011). However, neither the bacterial nor archaeal *amoA* genes responsible for the first step in this process (Boyd et al., 2011) were detected in the metagenome. Genes associated with anammox (hydrazine synthase and hydrazine dehydrogenase, Kartal et al., 2011) were also not detected. The SSU rRNA gene data for BF09-06 does not contain any Planctomycetes, which are the only lineage demonstrated to be able to carry out anammox to date, although there are Planctomycetes present in the spring water. Both these metabolisms could be present at a very low level, with gene numbers too small to be detected by the depth of our sequencing, but it is surprising not to find either one or the other. The best hypothesis to explain the lack of ammonium oxidation is that the bulk samples required for geochemical analysis and DNA extraction in this low biomass system may have sampled across any stratification in chemical species. This could mean that the ammonium was not present in the same part of the deposit as either nitrite or oxygen, so that neither aerobic nor anaerobic oxidation of ammonium could occur. However, the deposit was a paste-like consistency and so diffusion of chemical species should be possible. In environments where anammox is known to take place, such as the Black Sea, a stratified system has been observed. Oxidation of ammonium by nitrite occurs in anoxic zones, with nitrite introduced by mixing or diffusion from more oxidized zones nearer the surface, and with potential overlap of both anammox and nitrification in suboxic zones (Thamdrup and Dalsgaard, 2002; Kuypers et al., 2003; Dalsgaard et al., 2005; Lam et al., 2007).

### CONSTRAINTS

There are several factors that constrain our findings. The genome coverage data (**Table 4**) demonstrates that the microbial community has not been fully sampled, and so it is possible that some of the “missing metabolisms” are present at such a low abundance that they are below the level of detection. Even if this is the case, we still consider it significant that the relative abundance of several key genes is so low for reactions that are energetically significant. The second important constraint is that, as with all molecular studies, we can only identify genes that are closely related to annotated sequences in public databases. If the best hits for our sequences are GenBank sequences that have been mis-annotated, then this would affect our results. As we describe above, mis-annotation may explain the unexpectedly low abundance of *soxA* and *soxX* genes. However, mis-annotation does not explain the lack of phototrophic or ammonium oxidation genes. The reason that there was a significant impact on the *soxA* and *soxX* genes was because the *Sulfurovum* sp. and *Sulfuricurvum* sp. are the numerically dominant members of this community. In addition, it was only these two genes that appeared to be affected and other sulfur redox genes were detected in high abundance. The mis-annotation of a gene from low abundance organisms would not have such a significant impact on the result. A related constraint is that we cannot include genes of unknown function in our analysis. Finally we have considered whether read length could bias our results, and we do not consider this to be a factor. The metagenome library was produced from a shotgun library using a standard method that is totally independent of the DNA sequence. The only amplification step in the process is the one that occurs during the sequencing run itself. As a result, read length is unrelated to gene length, gene function, or taxonomic origin. While it is harder to accurately assign function and taxonomic origin to the shorter reads within our library, we would expect the shorter reads to be randomly distributed across genes of different function and different taxonomic origin, so we do not consider it likely that our results are impacted by any functional or taxonomic bias.

## CONCLUSION

Our study has raised new questions about the environmental factors that determine the success of different microbial metabolisms. Our results demonstrate potentially habitable niches that appear to be unoccupied. The relative absence of photosynthesis is particularly striking given the presence of abundant light and the reasons for this absence have not been determined. This finding has implications for understanding the environmental constraints that may impact photosynthesis in other systems. We have also discovered the Epsilonproteobacteria *Sulfurovum* and *Sulfuricurvum* to be numerically dominant in a sub-aerial setting for the first time. This suggests that they may have a wider distribution, and greater impact on sulfur cycling, than has previously been observed. In addition, this study is not only relevant to our understanding of the environmental factors that impact habitability in environments on Earth, but also adds to our understanding of how to assess the potential habitability of icy, sulfur-rich sites on Mars, Europa, or other planetary bodies.

## Conflict of Interest Statement

The authors declare that the research was conducted in the absence of any commercial or financial relationships that could be construed as a potential conflict of interest.

## Footnotes

1 http://sourceforge.net/projects/microbiomeutil/files/

2 http://www.ncbi.nlm.nih.gov/

3 http://metagenomics.anl.gov/
